# Detecting anti–SARS-CoV-2 antibodies in urine samples: A noninvasive and sensitive way to assay COVID-19 immune conversion

**DOI:** 10.1126/sciadv.abn7424

**Published:** 2022-05-13

**Authors:** Fernanda Ludolf, Fernanda F. Ramos, Flávia F. Bagno, João A. Oliveira-da-Silva, Thiago A. R. Reis, Myron Christodoulides, Paula F. Vassallo, Cecilia G. Ravetti, Vandack Nobre, Flavio G. da Fonseca, Eduardo A. F. Coelho

**Affiliations:** 1 Programa de Pós-Graduação em Ciências da Saúde, Infectologia e Medicina Tropical, Faculdade de Medicina, Universidade Federal de Minas Gerais, Belo Horizonte 30.130-100, Minas Gerais, Brazil.; 2 Laboratório de Virologia Básica e Aplicada, Departamento de Microbiologia, Instituto de Ciências Biológicas, Universidade Federal de Minas Gerais, Belo Horizonte 31.270-901, Minas Gerais, Brazil.; 3 Centro de Tecnologia em Vacinas (CT Vacinas), BH-Tec, Universidade Federal de Minas Gerais, Belo Horizonte 30.130-100, Minas Gerais, Brazil.; 4 Neisseria Research Group, Molecular Microbiology, School of Clinical and Experimental Sciences, University of Southampton Faculty of Medicine, Southampton General Hospital, Southampton SO16 6YD, UK.; 5 Hospital das Clínicas, Universidade Federal de Minas Gerais, Belo Horizonte 30.130-100, Minas Gerais, Brazil.; 6 Departamento de Clínica Médica, Faculdade de Medicina, Universidade Federal de Minas Gerais, Belo Horizonte 30.130-100, Minas Gerais, Brazil.; 7 Departamento de Patologia Clínica, COLTEC, Universidade Federal de Minas Gerais, Belo Horizonte 31.270-901, Minas Gerais, Brazil.

## Abstract

Serum-based ELISA (enzyme-linked immunosorbent assay) has been widely used to detect anti–severe acute respiratory syndrome coronavirus 2 (SARS-CoV-2) antibodies. However, to date, no study has investigated patient urine as a biological sample to detect SARS-CoV-2 virus-specific antibodies. An in-house urine-based ELISA was developed using recombinant SARS-CoV-2 nucleocapsid protein. The presence of SARS-CoV-2 antibodies in urine was established, with 94% sensitivity and 100% specificity for the detection of anti–SARS-CoV-2 antibodies with the urine-based ELISA and 88% sensitivity and 100% specificity with a paired serum-based ELISA. The urine-based ELISA that detects anti–SARS-CoV-2 antibodies is a noninvasive method with potential application as a facile COVID-19 immunodiagnostic platform, which can be used to report the extent of exposure at the population level and/or to assess the risk of infection at the individual level.

## Teaser

Urine sampling offers a new, viable, and reliable noninvasive possibility for detecting anti–SARS-CoV-2 antibodies.

## INTRODUCTION

The outbreak of coronavirus disease 2019 (COVID-19) caused by infection with SARS-CoV-2 (severe acute respiratory syndrome coronavirus 2) spread rapidly throughout the world, and in just 3 months, it was declared as a pandemic (*1*). This outbreak has highlighted the importance of readily available diagnostic tests to contain emerging and reemerging diseases. Diagnosis of COVID-19 can be done by direct detection of either SARS-CoV-2 RNA or SARS-CoV-2 antigens, as well as by indirect detection of specific antibodies (serological assays) (*2*–*5*). These strategies play different roles in distinct settings, from point-of-care testing to large-scale epidemiological surveillance (*4*). However, independently of the used tests, it is important to understand their performance, characteristics, and limitations to use them properly (*6*).

Many different serological assays are available for COVID-19, including the enzyme-linked immunosorbent assay (ELISA). These tests rely on the use of SARS-CoV-2 proteins to assess the presence of host-specific antibodies, such as immunoglobulin G (IgG), IgM, and IgA. Serological tests can identify individuals who have developed immunity to SARS-CoV-2 approximately 2 weeks post symptoms onset (PSO), and this information contributes substantially to epidemiological studies by helping to determine previous exposure to SARS-CoV-2 on an individual and/or population level (*4*, *5*).

Drawing blood for serological tests is one of the most common invasive procedures in health care, and although it has a low rate of complications, it can be unpleasant and difficult to perform in some circumstances. Collecting blood from children can be difficult, and even some adults might not wish to provide blood samples because of religious and/or personal reasons. The procedure also requires a trained phlebotomist, who may potentially be exposed to blood-borne pathogens. Blood collection via venipuncture can be especially challenging in some environments such as rural areas with limited health care resources and access (*7*, *8*). Dried blood spots collected by finger or heel prick are a minimally invasive alternative with the potential to solve some of the logistical challenges associated with venipuncture and offer the advantages of sample stability and the possibility of self-collection (*9*). Urine is a potentially useful sample for serological testing because collection is noninvasive, simple, and safe, and urine is easy to handle and store and very convenient for the individual and for clinical practice.

It has been known since the mid-1950s that γ-globulins can be detected in urine (*10*, *13*). Although not widely studied or reported, urine-based diagnostic tests that detect antibodies have been suggested as a possible noninvasive alternative to diagnose several conditions, such as dengue, *Helicobacter pylori* infection, hepatitis A and C, human immunodeficiency virus, strongyloidiasis, schistosomiasis, paragonimiasis, and leishmaniasis (*11*–*20*). To the best of our knowledge, there is no published study on detecting anti–SARS-CoV-2 antibodies in urine. In the present study, we used a recombinant (r)SARS-CoV-2 nucleocapsid (N) protein as part of an in-house ELISA to examine the presence of antiviral antibodies in urine samples collected from patients with SARS-CoV-2 infection, which was confirmed previously by quantitative reverse transcription polymerase chain reaction (qRT-PCR).

## RESULTS

We customized an in-house urine-based ELISA protocol using rSARS-CoV-2 N protein to evaluate the presence of anti–SARS-CoV-2 antibodies in the urine of patients with SARS-CoV-2 infection that had been confirmed previously by qRT-PCR. We used the original and well-established serum-based ELISA to compare accuracy and to validate our data. We included 139 adult hospitalized or nonhospitalized patients in this study (Table 1), only after positive confirmation of SARS-CoV-2 infection by qRT-PCR. Hospitalized patients recruited for this study accounted for 16.3% of the total number of COVID-19–confirmed patients admitted during the collection period at the study hospitals. For this study, we collected 209 urine and 187 serum paired samples, which varied from the 2nd to the 60th day PSO. We also included unpaired negative samples collected before 2019 and from individuals who had maintained a rigorous quarantine regimen and did not show any symptoms of COVID-19.

**Table 1.** Demographic breakdown of the study cohort. ICU, intensive care unit.

**Characteristics**	**Healthy subjects (*n* = 30)**	**Nonhospitalized patients (*n* = 11)**	Hospitalized patients^*^ (*n* = 128)
Age, mean (±SD)	40.5 ± 16.6	45.1 ± 5	62 ± 21
Female sex, %	75	45.5	41.8
Ward, %	0	0	29.75
ICU admission, %	0	0	70.35

*Hospitalized patients recruited for this study accounted for 16.3% of the total number of COVID-19–confirmed patients admitted during the collection period at the study hospitals.

### Detection of specific SARS-CoV-2 antibodies in urine samples

From a total of 209 urine samples collected from 139 qRT-PCR–positive patients, at different days PSO, we found that 187 urine samples reacted with the rSARS-CoV-2 N protein above the positive index value of >1.1, whereas 15 samples were classified as “indeterminate” (index values from 0.8 to 1.1) and 7 had a negative index value (<0.8) (Table 2). In addition, nonhospitalized individuals with mild symptoms (*n* = 11) showed positive (*n* = 9) or indeterminate (*n* = 2) index values, all of which were ≥0.9 (table S1). None of the urine samples obtained from pre– and post–COVID-19 negative controls (*n* = 19 and *n* = 11, respectively) reacted with the rSARS-CoV-2 N protein with a positive index above 1.1. Moreover, 26 samples had a negative index value below 0.8 and 4 had an indeterminate index value of 0.81, 0.85, 0.87, or 0.94 (Table 2).

**Table 2.** Evaluation of the presence of anti–SARS-CoV-2 antibodies in urine samples.

	Positive sample^*^	**Negative sample^†^**
Positive index (>1.1)	187 (90%)	-
Indeterminate index (0.8 to 1.1)	15 (7%)	4 (13%)
Negative index (<0.8)	7 (3%)	26 (87%)
**Total samples**	**209 (100%)**	**30 (100%)**

*Positive samples are collected from 139 qRT-PCR–positive patients, in different days PSO.

†Negative samples collected before 2019 were considered truly negative and called “pre–COVID-19.” Negative samples from individuals who had maintained a rigorous quarantine regimen and did not show any symptoms were considered theoretically negative and called “post–COVID-19.”

### Evaluation of IgG immunological conversion

**Fig. 1.**
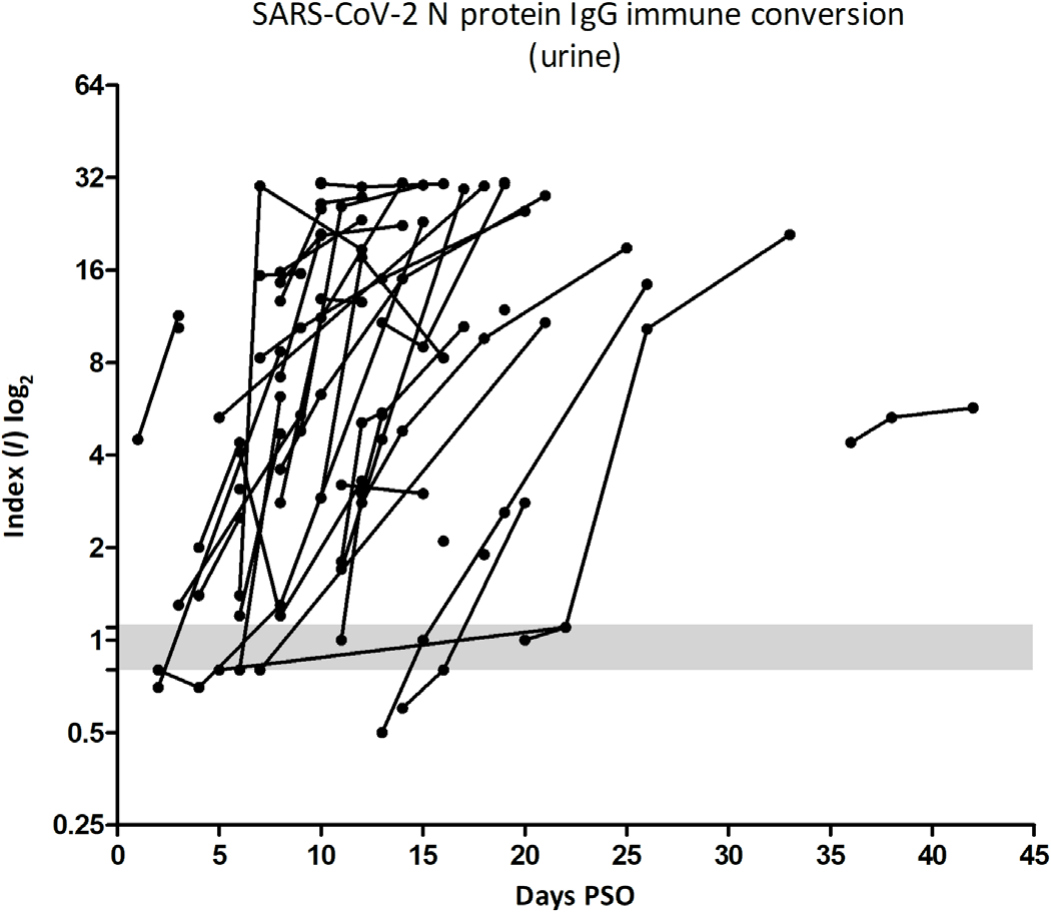
IgG immunological conversion to SARS-CoV-2 N protein in patient urine samples. Patients are represented by the lines interconnected by the points (sample collected in different days PSO). The immunological conversion observed for each patient varied in days PSO. The plotted index values (*I*) are related to the absorbance ratio of the cutoff values. Positive index value, above 1.1; indeterminate index value, between 0.8 and 1.1 (gray); and negative index value, below 0.8.

We did a serial evaluation of IgG antibody in urine and serum samples from 44 patients on days 1, 3, 7, and 14 after their inclusion in the study. Immune conversion for SARS-CoV-2 N protein was observed in urine, with an increase in IgG levels after symptoms onset, which varied depending on the patient (Fig. 1). A comparative ELISA using either urine or serum samples, from 34 patients, with at least 2 days of sample collection, revealed that immune conversion takes places with small variation between the different samples. However, we observed higher index values for the urine samples (figs. S1, A to D). For patients from whom only one sample was collected, it was not possible to determine the PSO day when immune conversion occurred, and thus, it was only possible to determine whether they were positive or negative on the day of sample collection.

### Immune conversion distribution of hospitalized patients by days PSO

**Fig. 2.**
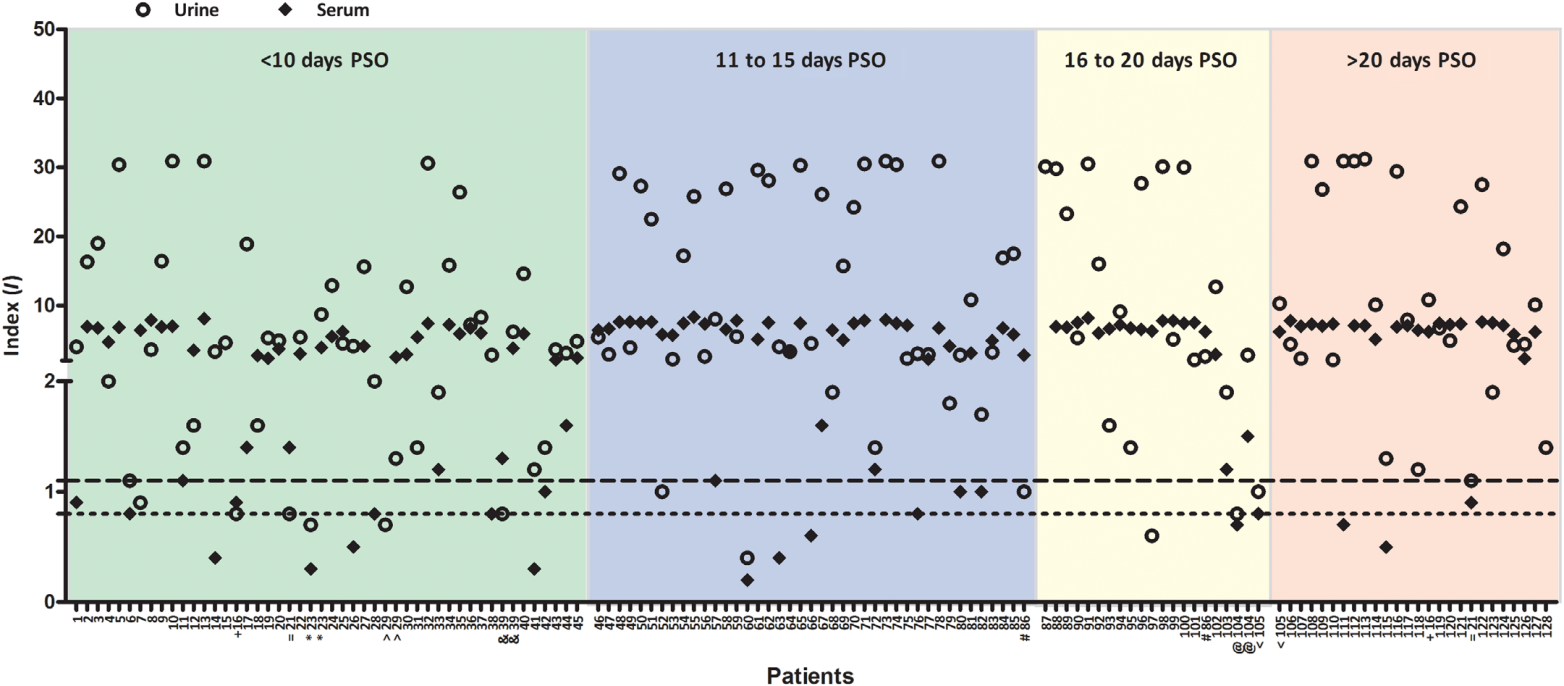
Urine and serum index values (*I*) of each patient according to the days PSO. The index values obtained from urine and serum samples for each patient are represented by circles and diamonds, respectively. Both samples, from the last day before immune conversion (index < 1.1) and from the first day of immune conversion (index > 1.1), collected from the same patient were plotted and are indicated by the matching symbol below the patient number. Only the first sample collected was plotted for those patients with all samples with positive index. Individual data were divided according to the PSO days of the collection date: <10 (green), 11 to 15 (blue), 16 to 20 (yellow), and >20 days (red). Positive index value, above 1.1; indeterminate index value, between 0.8 and 1.1; and negative index value, below 0.8.

A distribution plot was generated based on the PSO day of each patient’s sample collection to assess the time window when most patients reported a positive index value, thus indicating the presence of specific antibodies. For individuals with more than one collection, we plotted only samples from the first collection with a positive index value. From a total of 128 patients, 123 patients (96%) showed positive index values (>1.1) for their urine samples collected before 60 days PSO. From a total of 125 patients, 107 patients (86%) showed a positive index value (>1.1) for their paired collected serum samples. Four patients had a negative index value (<0.8) for their urine samples, while eight patients had a negative index value for their serum samples, collected before 20 days PSO. After 20 days PSO, no patient had a negative index value (<0.8) for their urine samples, whereas two patients had a negative index value for their serum samples (Fig. 2).

A possible immune conversion window, based on the day of PSO, could be determined for some patients with more than two urine collections, who obtained a negative index value (<0.8) for the first sample collection and became positive (index > 1.1) on subsequent collections. Five patients (patients 23, 29, 39, 86, and 104) turned positive (index > 1.1) before 20 days PSO and two patients (patients 16 and 105) after 20 days PSO, while only one (patient 21) maintained an indeterminate index value (from 0.8 to 1.1) after 20 days PSO, as highlighted by the paired symbols located below the patient number in Fig. 2.

### Comparing the accuracy of urine- and serum-based ELISA

**Fig. 3.**
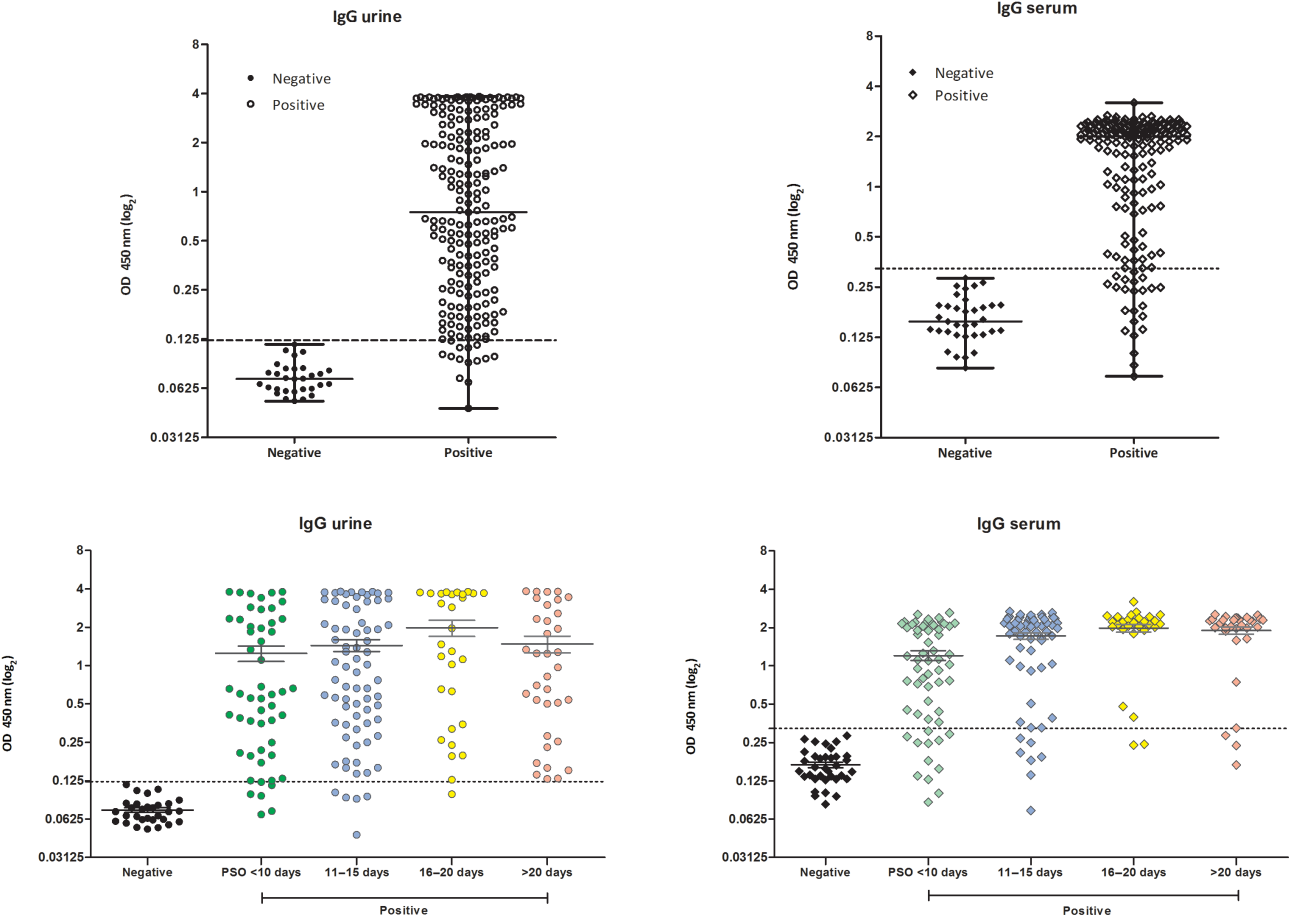
Evaluation for SARS-COV-2 diagnosis by using rSARS-CoV-2 N protein against patient urine and serum samples. ELISA was done using urine and serum samples (*n* = 209 and *n* = 187, respectively) from COVID-19 patients with positive qRT-PCR. Urine and unpaired serum samples from healthy subjects (*n* = 30 and *n* = 37, respectively) were also used. The mean of each group is shown, and the dashed line indicates the cutoff value determined for each type of biological sample (urine = 0.123 and serum = 0.323). The cutoff values were determined as the mean plus three times the SD of negative samples. Bottom: Positive sample groups are divided according to the PSO days of the collection date: <10 (green), 11 to 15 (blue), 16 to 20 (yellow), and >20 days (red).

**Fig. 4.**
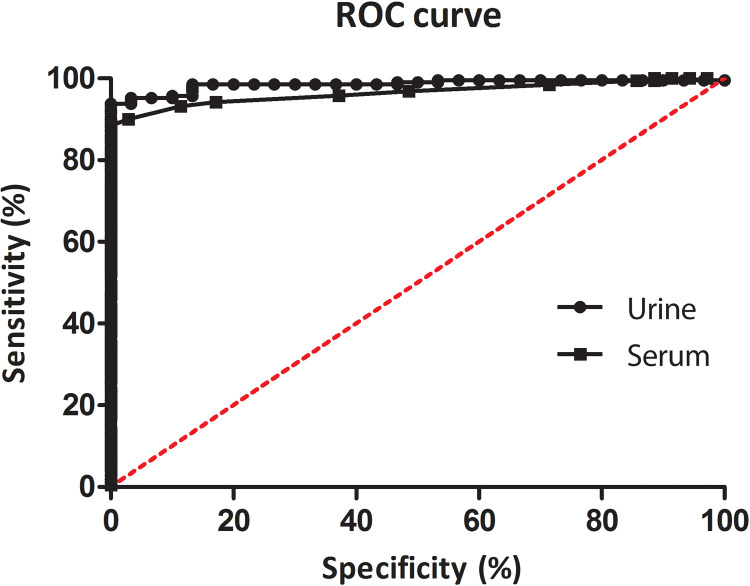
ROC curve for comparative diagnostic performance of urine- and serum-based ELISA for COVID-19. ROC curves were constructed using the individual OD values for each sample to obtain the sensitivity, specificity, and area under the curve.

We evaluated the diagnostic efficacy for COVID-19 of the rSARS-CoV-2 N protein by ELISA against a panel of urine and paired serum samples, collected at the same time, from hospitalized and nonhospitalized patients. For the analyses, urine and serum (*n* = 209 and *n* = 187, respectively) samples from qRT-PCR–positive patients were used, as well as unpaired negative samples from pre–COVID-19 (*n* = 19) and post–COVID-19 (*n* = 11) urines and pre–COVID-19 (*n* = 30) and post–COVID-19 (*n* = 5) sera. The individual OD (optical density) values determined for each urine or serum sample against the rSARS-CoV-2 N protein are shown in Fig. 3. Sensitivity and specificity values of 93.81 and 100%, respectively, were calculated for urine samples tested in ELISA, as well as 87.70 and 100%, respectively, for serum samples. Comparative diagnostic performance of urine- and serum-based ELISA for COVID-19, under optimal experimental protocols for each biological specimen, is presented in Table 3. Receiver operating characteristic (ROC) curves showed marginally superior accuracy when urine was tested (*R* value = 0.9856) compared to serum (*R* value = 0.9577), but this was not statistically significant (Fig. 4).

**Table 3.** Comparative IgG anti–SARS-CoV-2 N protein diagnostic performance of the in-house urine- and serum-based ELISA. The diagnostic performance of the antigen against the urine and serum samples was based on the estimation of sensitivity (Se), specificity (Sp), area under the curve (AUC), 95% confidence level (95% CI), and Youden index (*J*). Positive and negative predictive values (PPV and NPV, respectively) were calculated on the basis of the index value, excluding the indeterminate value samples, and using the following equations: NPV = true negative/false negative + true negative and PPV = true positive/false positive + true positive. CI, confidence interval.

**Sample**	**AUC**	***P* value**	**Cutoff**	**Se (%)**	**95% CI**	**Sp (%)**	**95% CI**	***J***	**PPV**	**NPV**
**Urine**	0.9856	<0.0001	0.123	93.81	89.65–96.66	100	88.06–100	0.94	1.0	0.79
**Serum**	0.9577	<0.0001	0.323	87.70	82.12–92.04	100	90.00–100	0.88	1.0	0.70

## DISCUSSION

In the present study, we compared the ability of an rSARS-CoV-2 N protein–based ELISA to discriminate serologically positive patients with COVID-19 using either urine or serum samples. Our data showed sensitivity and specificity values of 94.0 and 100%, respectively, when urine was used, and the presence of antibodies in patients’ urine was observed a few days PSO. The use of urine gave higher sensitivity to detect seroconversion over the use of sera under optimal experimental conditions. Serum-based ELISA tests have been used to diagnose SARS-CoV-2 seropositivity by detecting specific antibodies in patient serum samples (*4*). However, to the best of our knowledge, this is the first study using a noninvasive urine-based ELISA test to identify specific antibodies against a SARS-CoV-2 protein. The use of urine to detect antibodies could be considered more convenient for clinical practice and for epidemiological surveillance compared to the challenges encountered with venipuncture, because it (i) allows patients to collect their own samples and (ii) eliminates the need for trained phlebotomists to draw blood and the attendant risks involved in handling potential blood-borne pathogens (*11*). We used sodium azide as a preservative for this study, as the presence of detectable antibodies in urine samples stored at 4°C has been reported for as long as 6 years (*14*). Nonetheless, the stability of five refrigerated urine samples without sodium azide was examined for five consecutive days, and our data showed that there was no change in assay performance between fresh and stored samples (fig. S2).

SARS-CoV-2 has four structural proteins—spike (S), envelope (E), membrane (M), and nucleocapsid (N) proteins—and all of them have already been tested as antigens for COVID-19 diagnosis using recombinant proteins, synthetic peptides, and/or polypeptide chimeras (*4*, *6*, *22*). Although many diagnostic tests use the recombinant protein S as the antigen, ELISA-based assays using a recombinant N protein have also been widely used with high sensitivity to detect antibodies in mildly infected patients (*21*, *23*). In the current study, we found that the diagnostic performance of the serum-based ELISA (sensitivity and specificity of 87.7 and 100%, respectively) was marginally lower than that of the urine-based ELISA using recombinant N protein.

Specific IgG SARS-CoV-2 seroconversion usually occurs at 7 to 14 days after infection, and it is presumably accompanied by the development of protective immunity (*4*). In the early stages of infection, commercial serum-based antibody tests show low accuracy because most of the patient’s immune response is still developing. The accuracy of serological tests can be near 100%, when samples are acquired at 20 days PSO (*4*, *8*, *24*–*26*). Note that urine samples collected from positive qRT-PCR patients who did not demonstrate positive index values might have been collected too early for seroconversion to occur. We confirmed this with a longitudinal analysis of the samples, collected from the same patients on different days PSO, who turned positive along the course of the disease (fig. S1). In our study, immune conversion for urine and serum occurred at a similar rate, with an increase in IgG production along the days PSO, when individual index values were plotted for such samples (fig. S1). ELISA-positive index values were obtained with many of the samples collected before 10 days PSO, at least for the hospitalized individuals. Nonetheless, we cannot assume at this moment that early detection and more rapid rise of antibodies would be due to a first or prior SARS-CoV-2 infection, as such information is lacking. Conversely, few samples showed a negative index value even after 10 days PSO collection.

We identified antibodies in patients up to 60 days PSO; however, a longer follow-up study is still necessary to establish the diagnostic detection window of anti–rSARS-CoV nucleocapsid antibodies in urine samples. The identification of anti–hepatitis A virus antibodies in urine appeared to be comparable to serum for the diagnosis of recent and past infection and could last for as long as 130 days PSO (*15*). It is important to be aware that a distinct diagnostic detection window may be found for urine- and serum-based ELISA. This difference is found for visceral leishmaniasis (VL), for which the detection of specific antibodies in serum persists after cure, while the presence of antibodies completely disappears after 6 months when using a urine-based ELISA. This observation indicates a possible use for the specific diagnosis of VL and also for monitoring the treatment response (*20*).

Our study has suggested similar patterns of baseline antibody responses for urine-based ELISA to those found for serum-based ELISA and with substantial variation in the magnitude of the responses between participants within each severity category, hospitalized or nonhospitalized (*27*). The degree and duration of immunity that antibodies confer, from infection or vaccination, remain unclear (*28*). Notably, if IgG antibodies are present, they often indicate a previous infection, but do not exclude an ongoing infection.

Vaccines approved to protect against SARS-CoV-2 infection and administered around the world, such as Oxford/AstraZeneca adenovirus viral vector AZD1222, Pfizer-BioNTech mRNA vaccine BNT162b2, Johnson & Johnson’s Janssen modified viral vector, Moderna mRNA Spikevax, and Sputnik V adenovirus viral vector, use only the S protein of SARS-CoV-2 to elicit protective immunity. For these vaccines, the SARS-CoV-2 N protein antibody response evaluated by ELISA in our study could be used potentially as a reliable tool for assessing antibody responses to COVID-19 versus antibody responses induced by vaccination. On the other hand, the inactivated CoronaVac/Sinovac or Sinopharm viral vaccines contain the N protein in their formulation and could be evaluated to confirm vaccine-induced antibody conversion. Because of the diversity of the vaccines licensed in each country and the types of immunological tests used (SARS-CoV-2 N and/or S proteins), humoral conversion results can be misinterpreted by patients who may not always know what type of antibody test was used (*29*, *30*).

Many individuals do not have their infectious status confirmed by the direct detection of either SARS-CoV-2 RNA or antigens, because of the limitations of tests, the short detection window, or the availability of tests to the patients. While antibody tests should not be used to establish the presence or absence of acute SARS-CoV-2 infection, they can be a useful tool for identifying people with resolving or past SARS-CoV-2 infection, which can aid diagnosis and complications arising from COVID-19 (*8*). In this sense, the development of our N protein urine-based ELISA becomes an additional available tool for individual and epidemiological use. Furthermore, because we have identified antibodies to the SARS-CoV-2 N protein in urine, the development of a urine-based spike ELISA test may also be feasible to cover other applications of serological tests such as the detection of vaccine-induced antibodies.

Urine collection has been neglected as a biological specimen because urine-based serological tests are uncommon. Our study included 19 urine samples collected before the outbreak in 2019, and all of them showed index values of <1.1. We also included 11 urine samples collected during 2021 from individuals who remained in quarantine and who did not show any symptoms throughout 2020/2021, although they cannot be considered as true negatives. Nevertheless, all these samples also showed an index of <1.1 (no positive).

A minimum of 98% specificity was stated in some countries for lateral flow immunoassay home tests, as false-positive results may increase the risk of self-exposure of nonimmune protected individuals (*31*). ELISA is an analytical method used to identify and measure analytes in low concentrations with less risk of interference. As a quantitative and qualitative assay, its cutoff is defined to distinguish between positive and negative samples with accuracy (*32*). The N protein was specific for SARS-CoV-2 in our ELISA, because we observed no humoral reactivity in serum samples from patients with respiratory tract viral infections (influenza, measles, and parvovirus), patients with arboviruses (yellow fever virus, chikungunya, dengue, and Zika), and individuals vaccinated against influenza (30 to 60 days) (*21*). Nonetheless, our study has limitations, as we have not tested our ELISA against samples obtained from patients with respiratory infections caused by other types of coronavirus. Therefore, we cannot rule out the possibility of cross-reactivity with other human coronaviruses.

The comparative use of paired serum samples as reference was fundamental for our study because of their conventional use in ELISA, noting that Bagno *et al.* (*21*) had already validated the serum-based ELISA platform used here. Urine-based and serum-based ELISA achieved a very similar qualitative profile. However, we should interpret the quantitative comparison of reaction intensity between samples with care, as urine samples were collected without a fixed standard retention time; thus, there may be variation in antibody concentration. It would be important in a future study to determine and fix retention time, as well as for its use in clinical practice. When the effect of the timing of urine collection was studied for *Wuchereria bancrofti* infection, with urine collections in the early morning and later throughout the day, only a small fluctuation in antibody units was observed and all samples remained positive (*33*, *34*). In a urine-based ELISA study conducted for *H. pylori*, it was observed that the urine water content influenced the concentration of antibody in urine; however, the performance of the qualitative assay on post-fasting urine correlated well with those of serum samples (*33*, *35*). Various features of urine, beyond retention time, could influence signal strength relative to serum and deserve future investigation. Changes in urine pH and the presence of microorganisms had no significant effect on the absorbance value of the *H. pylori* urine-based ELISA. Moreover, sodium azide is commonly added to prevent changes in urine pH resulting from contamination and growth of bacteria (*33*, *35*). Patients with significant proteinuria should be tested with caution as demonstrated for the *H. pylori* urine-based ELISA (*35*).

In summary, our preliminary study suggests that a urine-based ELISA using a recombinant N protein could be useful for diagnosing SARS-CoV-2 immune conversion. Advantages of the assay included ease of sample collection, biological sample stability, easy assay standardization, and high levels of accuracy. This noninvasive immunoassay can become a useful tool to guide public health policy across the clinical and research environment, with effects to be provided at the individual and population level.

## MATERIALS AND METHODS

### Experimental design

First, we evaluated the presence of anti–SARS-CoV-2 nucleocapsid-specific antibodies in 209 urine samples, collected between the 2nd and 60th PSO day, from 139 infected patients previously confirmed with qRT-PCR. We then checked the immune conversion window of 44 patients with follow-up collections on days 1, 3, 7, and 14 after their inclusion in the study. We used the original and well-established serum-based ELISA to compare accuracy and validate our findings. To assess the detection window time on which most patients found a positive index value, we plotted the PSO day distribution of the first day of positive urine and serum sample collection of 128 and 125 patients, respectively. Last, we compared the diagnostic accuracy of the rSARS-CoV-2 N protein against a panel of 209 urine and 187 serum paired samples, which we collected at the same time, between the 2nd and the 60th day PSO, from hospitalized and nonhospitalized patients. We also included unpaired negative samples collected before 2019 and from individuals who had maintained a rigorous quarantine and did not show any symptoms.

### Research subjects and biological samples

The study was approved by the Human Research Ethics Committee from the Federal University of Minas Gerais (UFMG; Belo Horizonte, Brazil) under protocol number CAAE 30437020.9.0000.5149. Patients seeking hospital assistance presenting respiratory symptoms were assessed by the attending physician and included in this study after confirmation of a positive qRT-PCR test for SARS-CoV-2. Nonhospitalized individuals who experienced mild COVID-19 symptoms and tested positive for SARS-CoV-2 by qRT-PCR were also included in this study. All included participants were adults, male or female, and signed an informed consent form. Hospitalized patients (*n* = 128) were recruited at Hospital das Clínicas of the UFMG (Belo Horizonte, Brazil) and Hospital Santa Helena (Betim, Brazil), and nonhospitalized individuals (*n* = 11) were recruited through active search in the general population (Belo Horizonte, Brazil).

Urine and serum samples from hospitalized patients were collected on the first day of inclusion and, whenever possible, on days 1, 3, 7, and 14 after recruitment, thus varying the corresponding day PSO for each patient. Urine and serum samples from nonhospitalized individuals, who tested positive for SARS-CoV-2 infection by qRT-PCR, were collected between 20 and 60 days PSO. Samples collected before 2019 were considered truly negative and called “pre–COVID-19.” Samples from individuals who had maintained a rigorous quarantine and did not show any symptoms were considered theoretically negative and called “post–COVID-19 negative.” Both sample sets were used as negative controls. The total number of urine samples used herein was 198 hospitalized and 11 nonhospitalized, and 19 and 11 pre– and post–COVID-19 negative, respectively. The total number of serum samples was of 226 hospitalized and 4 nonhospitalized, and 30 pre-COVID and 5 post-COVID negative.

Urine samples were collected any time of the day, using a 80-ml urine collection cup, when the liquid was transferred to 15-ml tubes containing 0.1% (w/v) sodium azide. Tubes were kept at room temperature for a few days or at 4°C for long time storage. The pre–COVID-19 urine samples were previously collected in 2018/2019 and kept at 4°C until use. Blood was collected in 20-ml tubes without anticoagulant, and serum was separated by centrifugation at 3000*g* for 15 min at 4°C and stored at −20°C until use.

### Enzyme-linked immunosorbent assay

ELISA was performed according to Bagno *et al*. (*21*) under optimal experimental conditions for urine or serum, as described below. Plates were coated with 0.4 μg per well of rSARS-CoV-2 N protein diluted in carbonate buffer for 18 hours at 4°C. After antigen binding, blocking was done with a solution of phosphate-buffered saline (PBS) containing 0.05% (v/v) Tween 20 (PBS-T) and 1% (w/v) bovine serum albumin for 2 hours at 25°C. Then, the wells were washed using PBS-T and incubated with 100 μl per well of urine (undiluted) or serum (1/100 dilution in PBS-T) samples for 1 hour or 30 min, respectively, at 37°C, when they were again washed. Using urine as samples, peroxidase-conjugated anti-human IgG antibody (Sigma-Aldrich), 1/10,000 dilution in PBS-T, was added, and the plates were incubated for 1 hour at 37°C. Using serum as samples, the conjugated antibody was diluted 1/80,000, and the plates were incubated for 30 min at 37°C. Next, the wells were washed, and reactions were developed by the addition of TMB (3,3′,5,5-tetramethylbenzidine) for 15 min in the dark. Reactions were stopped by adding 0.5 M H_2_SO_4_, and the OD values were read on a microplate spectrophotometer (Multiskan Go) at λ 450 nm. The cutoff values were determined as the mean plus three times the SD of negative samples. The index (*I*) value for each sample was calculated using the following equation: *I* = (OD λ450 nm)/(cutoff). The index value was classified positive above 1.1, indeterminate between 0.8 and 1.1, and negative below 0.8.

### Statistical analysis

Data collected from the included individuals were recorded in a dedicated form. The analyses were done using the GraphPad Prism program (version 8.0 for Windows). Value distributions (means ± SD, as indicated) were obtained for continuous variables, while categorical ones were evaluated as proportions. ROC curves were constructed with OD values of the positive versus negative (pre- and post-COVID negative) samples. The diagnostic performance was evaluated by estimation of sensitivity, specificity, area under the curve, and Youden index. Confidence intervals (CIs) were defined with 95% confidence level (95% CI). The paired *t* test was used to compare the distinct groups. *P* < 0.05 values were considered significant. Positive and negative predictive values (PPV and NPV, respectively) were calculated on the basis of the index value, excluding the indeterminate value samples, and using the following equations: NPV = true negative/false negative + true negative and PPV = true positive/false positive + true positive.

## Notes

View/request a protocol for this paper from *Bio-protocol*.
