# Supplementary material for: Detecting anti–SARS-CoV-2 antibodies in urine samples: A noninvasive and sensitive way to assay COVID-19 immune conversion
Source: Sci Adv. 2022 May 13;8(19):eabn7424. doi: 10.1126/sciadv.abn7424 (PMC9106288; doi:10.1126/sciadv.abn7424)
Supplement: Supplementary file 1 — Figs. S1 and S2 Table S1 [file sciadv.abn7424_sm.pdf]

Science Advances
AAAS

Supplementary Materials for

Detecting anti–SARS-CoV-2 antibodies in urine samples: A noninvasive and sensitive way to assay COVID-19 immune conversion

Fernanda Ludolf*, Fernanda F. Ramos, Flávia F. Bagno, João A. Oliveira-da-Silva, Thiago A. R. Reis, Myron Christodoulides, Paula F. Vassallo, Cecilia G. Ravetti, Vandack Nobre, Flavio G. da Fonseca, Eduardo A. F. Coelho

*Corresponding author. Email: feludolf@gmail.com

Published 13 May 2022, *Sci. Adv.* **8**, eabn7424 (2022)
DOI: 10.1126/sciadv.abn7424

This PDF file includes:

Figs. S1 and S2
Table S1

Supplementary material:

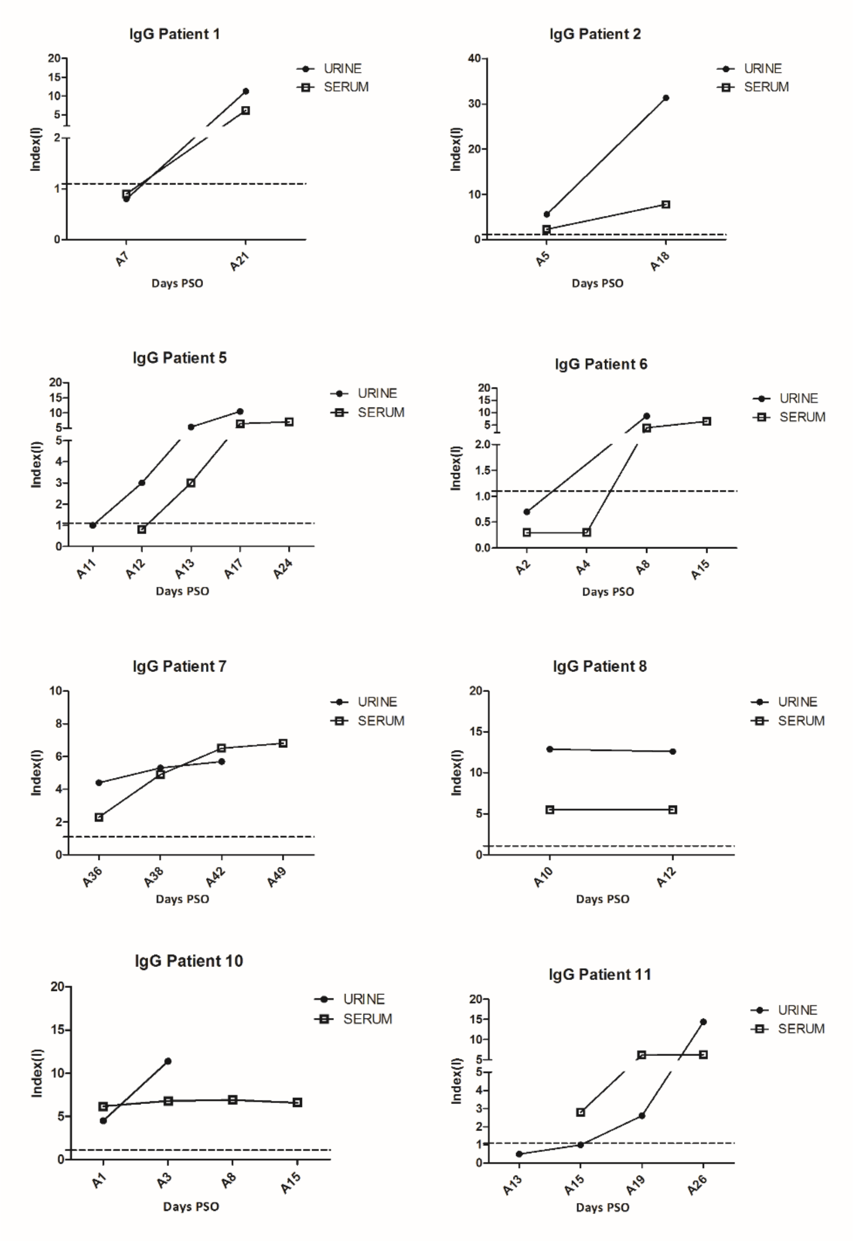

Fig. S1A. Dynamics of IgG antibody conversion in patient urine and serum samples. Graphs show the IgG levels specific to the rSARS-CoV-2 N protein using urine and serum samples from post-hospitalization patients, with collection on different days post-symptom onset. The plotted index values (I) were related to the absorbance ratio on the cut-off. Positive index value above 1.1 (dashed line), indeterminate between 0.8 and 1.1 and negative below 0.8.

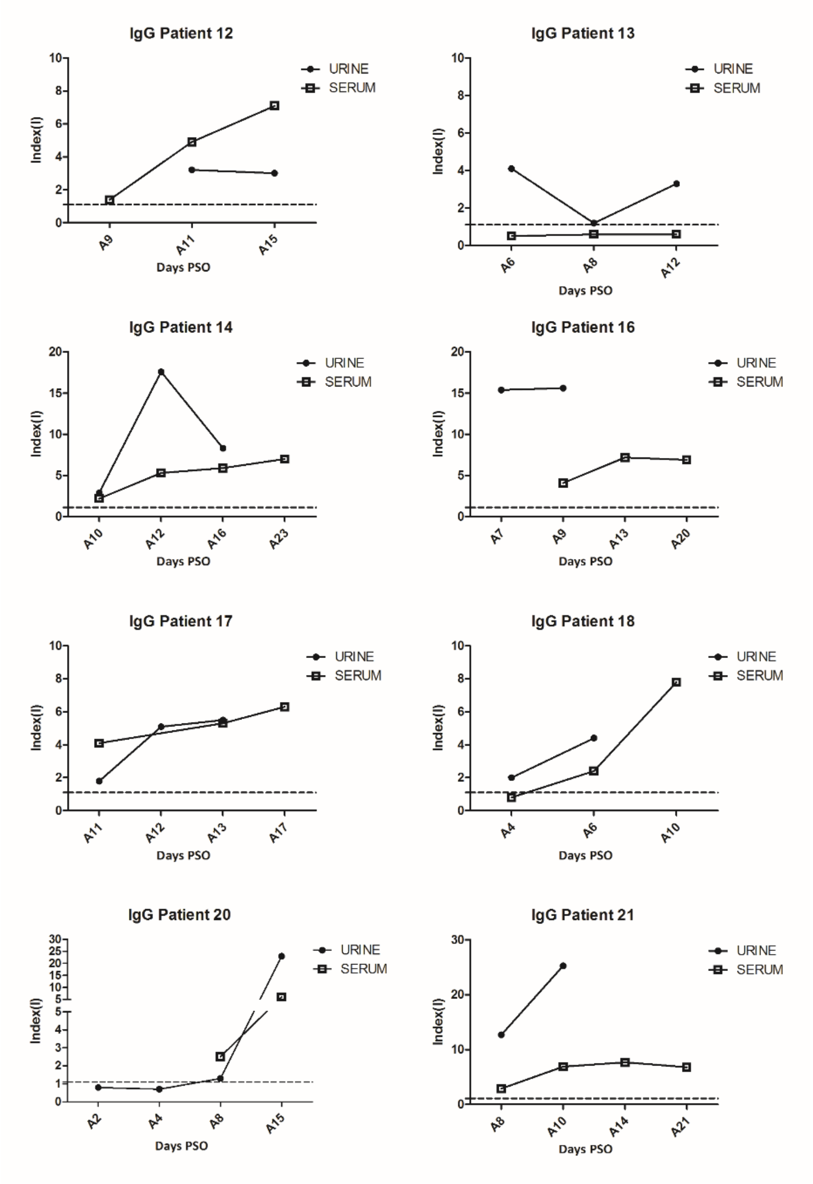

Fig. S1B. Dynamics of IgG antibody conversion in patient urine and serum samples. Graphs show the IgG levels specific to the rSARS-CoV-2 N protein using urine and serum samples from post-hospitalization patients, with collection on different days post-symptom onset. The plotted index values (I) were related to the absorbance ratio on the cut-off. Positive index value above 1.1 (dashed line), indeterminate between 0.8 and 1.1 and negative below 0.8.

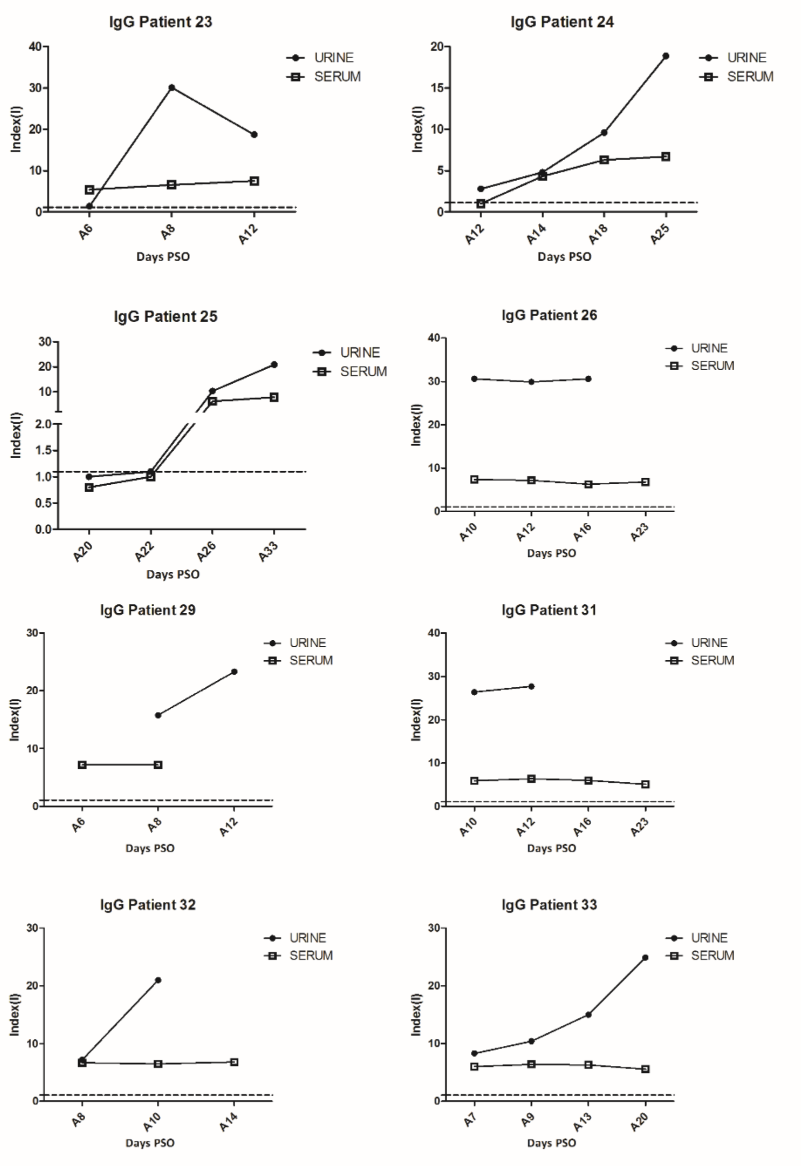

Fig. S1C. Dynamics of IgG antibody conversion in patient urine and serum samples. Graphs show the IgG levels specific to the rSARS-CoV-2 N protein using urine and serum samples from post-hospitalization patients, with collection on different days post-symptom onset. The plotted index values (I) were related to the absorbance ratio on the cut-off. Positive index value above 1.1 (dashed line), indeterminate between 0.8 and 1.1 and negative below 0.8.

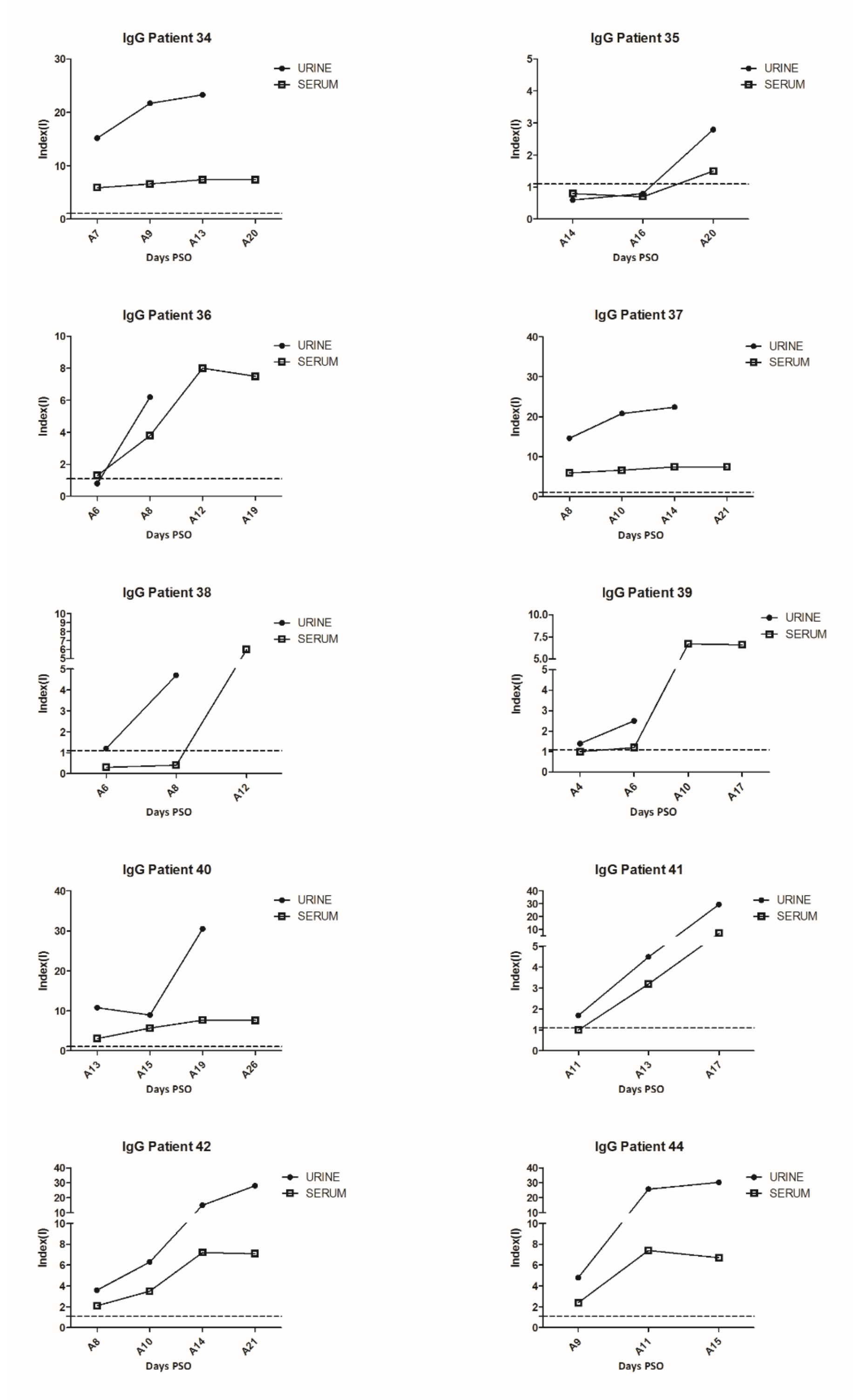

Fig. S1D. Dynamics of IgG antibody conversion in patient urine and serum samples. Graphs show the IgG levels specific to the rSARS-CoV-2 N protein using urine and serum samples from post-hospitalization patients, with collection on different days post-symptom onset. The plotted index values (I) were related to the absorbance ratio on the cut-off. Positive index value above 1.1 (dashed line), indeterminate between 0.8 and 1.1 and negative below 0.8.

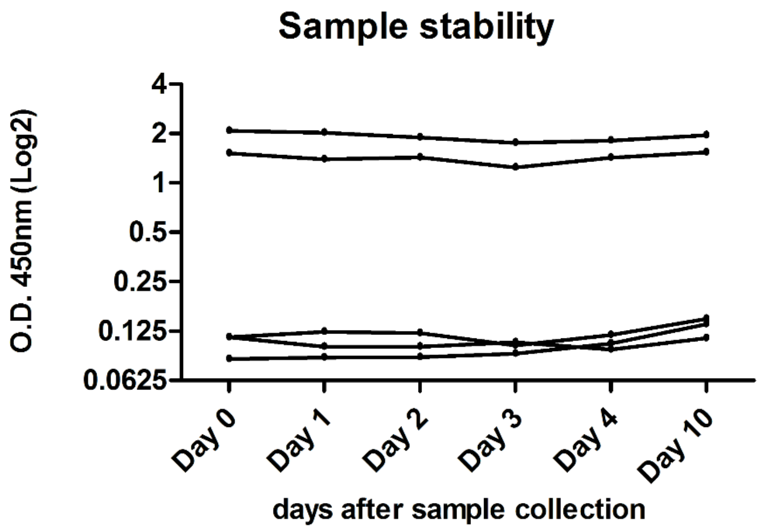

Fig. S2. Stability of urine sample by days after collection. The stability of five random urine samples, without sodium azide, was checked for 5 consecutive days and after 10 days. No change in assay performance was observed between fresh (day 0) versus refrigerated stored (days 1, 2, 3, 4 and 10) samples.

Table S1. Urine-based ELISA index values obtained for non-hospitalized patients, according to the day post symptoms onset (PSO).

Non-hospitalized individuals (n=11)	PSO (days)	Index (Value)*
1	20	2.5
2	60	11.3
3	30	1.5
4	30	0.9
5	60	1.4
6	21	1.1
7	46	3.4
8	59	2.0
9	60	0.9
10	25	1.7
11	30	1.2

Index (value): positive above 1.1, indeterminate between 0.8 and 1.1 and negative below 0.8.
